# Adjunctive Use of Botulinum Toxin in Orthognathic Surgery: A Systematic Review of Current Evidence and Clinical Perspectives

**DOI:** 10.3390/toxins18090399

**Published:** 2026-09-18

**Authors:** Audra Janovskienė, Justina Stučinskaitė-Maračinskienė, Žygimantas Petronis, Jan Pavel Rokicki, Dainius Razukevičius, Loreta Pilipaitytė

**Affiliations:** 1 Department of Maxillofacial Surgery, Medical Academy, Hospital of Lithuanian University of Health Sciences, Eiveniu 2, LT-50161 Kaunas, Lithuania; justina.stucinskaite-maracinskiene@lsmu.lt (J.S.-M.); zygimantas.petronis@lsmu.lt (Ž.P.); jan.pavel.rokicki@lsmu.lt (J.P.R.); dainius.razukevicius@lsmu.lt (D.R.); 2 Department of Plastic Surgery, Medical Academy, Hospital of Lithuanian University of Health Sciences, Eiveniu 2, LT-50161 Kaunas, Lithuania; loreta.pilipaityte@kaunoklinikos.lt

**Keywords:** botulinum toxin type A, BoNT-A, orthognathic surgery, skeletal relapse, skeletal stability, postoperative pain, bilateral sagittal split osteotomy

## Abstract

Background: Botulinum toxin type A (BoNT-A) has been proposed as an adjunct to orthognathic surgery because its temporary neuromuscular effects may reduce muscular loading during postoperative healing and potentially influence fixation stability, skeletal relapse, and postoperative recovery. This systematic review aimed to evaluate the available clinical evidence regarding the adjunctive use of BoNT-A in orthognathic surgery. Methods: A systematic literature search was conducted in PubMed, the Cochrane Library, and Google Scholar in accordance with the Preferred Reporting Items for Systematic Reviews and Meta-Analyses (PRISMA) guidelines. Clinical studies directly comparing orthognathic surgery with and without adjunctive BoNT-A administration and reporting outcomes related to mechanical stability, skeletal stability, or postoperative recovery were included. Risk of bias was assessed using the Cochrane Risk of Bias 2 (RoB 2) tool for randomized controlled trials and ROBINS-I for the non-randomized study. Results: Three studies met the eligibility criteria, comprising two randomized controlled trials and one retrospective comparative study. Considerable heterogeneity was observed in surgical procedures, targeted muscles, BoNT-A doses, timing of administration, and evaluated outcomes. Individual studies reported associations between BoNT-A administration and a lower incidence of fixation plate fracture, reduced anteroposterior skeletal relapse at the Pogonion, or lower preanalgesic pain scores and postoperative opioid consumption. However, these findings were derived from different studies evaluating distinct interventions and outcomes and were not consistently demonstrated across comparable outcome measures. Conclusions: BoNT-A may represent a promising adjunct to orthognathic surgery, with potential benefits for fixation-related mechanical stability, selected parameters of skeletal relapse, and postoperative pain management. However, the limited number of studies, methodological limitations, and substantial clinical heterogeneity preclude definitive conclusions. Further well-designed randomized controlled trials with standardized injection protocols and longer follow-up are required before routine clinical use can be recommended.

## 1. Introduction

Botulinum toxin type A (BoNT-A), a potent neurotoxin produced by the bacterium Clostridium botulinum, has been widely used for both therapeutic and aesthetic applications across various medical disciplines [1]. Its ability to induce temporary and reversible muscle relaxation has led to an expanding range of clinical indications, particularly in conditions associated with excessive or abnormal muscular activity. Following intramuscular injection, BoNT-A binds to presynaptic cholinergic nerve terminals and inhibits the release of acetylcholine at the neuromuscular junction. The resulting temporary chemical denervation reduces muscle contractile activity and induces reversible muscle relaxation [2,3,4]. These pharmacological properties have supported the use of BoNT-A in the management of various conditions associated with muscular hyperactivity and chronic pain, including migraine, myofascial pain, and temporomandibular disorders (TMDs) [5,6]. In addition to its neuromuscular effects, BoNT-A has suggested analgesic properties that may extend beyond muscle relaxation through the modulation of peripheral nociceptive signaling and a potential reduction in central pain sensitization.

Orthognathic surgery is performed to correct dentofacial deformities and restore functional and aesthetic relationships of the craniofacial complex [7]. Despite advances in surgical techniques, fixation methods, and perioperative management, postoperative complications and challenges, including skeletal relapse, fixation-related complications, pain, and delayed functional recovery, remain clinically relevant [8]. Postoperative skeletal relapse is a multifactorial process influenced by several anatomical and biomechanical factors, including bony interference, displacement of the proximal segment, improper condylar positioning, and tension generated by the surrounding soft tissues and masticatory muscles [9,10,11]. In particular, persistent muscular forces following skeletal repositioning may contribute to mechanical loading of fixation systems and influence postoperative skeletal stability. Although invasive procedures such as myotomy have previously been proposed to reduce unfavorable muscular tension, their associated morbidity has encouraged the investigation of less invasive approaches [12]. Given its ability to temporarily reduce muscle contractile activity, BoNT-A has emerged as a potential adjunctive intervention for modulating muscular forces following orthognathic surgery, with possible implications for mechanical and skeletal stability as well as postoperative recovery [13,14,15,16].

Despite the growing interest in BoNT-A applications within oral and maxillofacial surgery, clinical evidence regarding its adjunctive use in orthognathic surgery remains limited and heterogeneous. Existing studies have investigated different therapeutic targets and clinical outcomes, including the reduction in mechanical stress on fixation systems, prevention of postoperative skeletal relapse, and improvement of postoperative pain and recovery. Therefore, this systematic review aims to summarize and critically evaluate the current evidence regarding the adjunctive use of BoNT-A in orthognathic surgery, with particular emphasis on its potential role in mechanical stability, skeletal stability, and postoperative recovery. Additionally, this review seeks to identify current limitations in the available evidence and highlight areas requiring further investigation before BoNT-A can be considered for routine clinical application in orthognathic surgery.

## 2. Results

### 2.1. Study Selection

**Figure 1:**
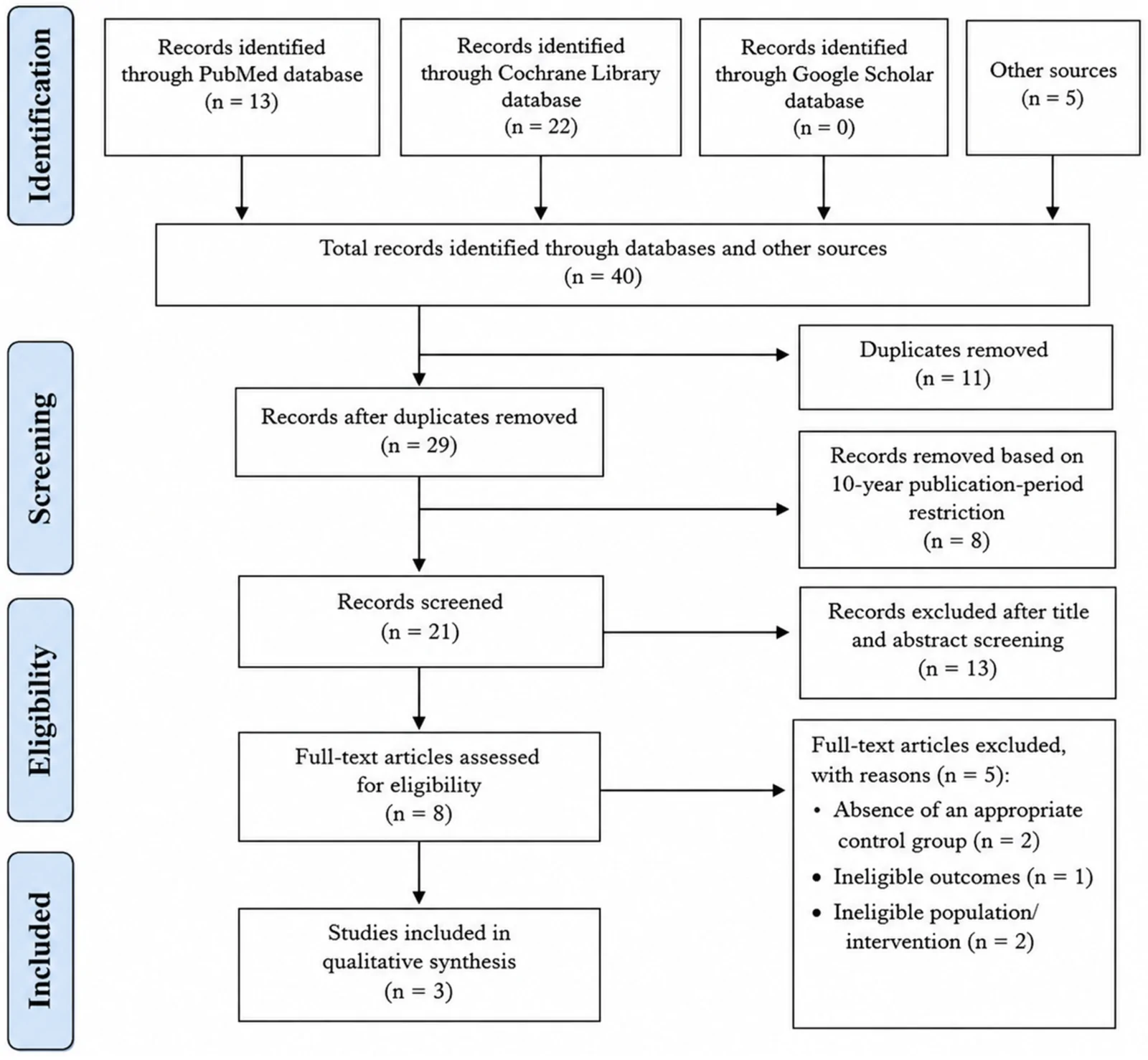
PRISMA flow diagram of the literature search and study selection process.

The initial database search identified 40 potentially relevant publications. After the removal of 11 duplicate records, 29 publications remained. Application of the predefined ten-year publication period restriction resulted in the exclusion of eight additional records, leaving 21 publications for title and abstract screening. Following the screening process, eight potentially eligible articles underwent full-text assessment. Of these, five studies were excluded based on the predefined eligibility criteria [17,18,19,20,21], resulting in three publications being included in the final systematic review. The complete study selection process is presented in the PRISMA flow diagram (Figure 1).

### 2.2. Risk of Bias Assessment

The risk of bias was assessed using tools appropriate to the design of the included studies. The two randomized controlled studies, Moutamed et al. [22] and Lung et al. [23], were evaluated using the Cochrane Risk of Bias 2 (RoB 2) tool. version 22 August 2019. Moutamed et al. [22] was judged to have some concerns regarding the overall risk of bias, primarily related to the randomization process, deviations from the intended interventions, outcome measurement, and selection of the reported results. Lung et al. [23] suggested a more robust methodological design; however, some concerns remained regarding the selection of the reported results, resulting in an overall judgement of some concerns.

As Shin et al. [24] conducted a non-randomized retrospective comparative study, risk of bias was assessed separately using the Risk Of Bias In Non-randomized Studies of Interventions (ROBINS-I) tool. The study was judged to have a serious overall risk of bias, primarily due to potential confounding associated with the non-randomized design and baseline differences between the intervention and control groups. A moderate risk of bias was additionally identified in the domains concerning participant selection, deviations from intended interventions, outcome measurement, and selection of the reported result, whereas the classification of interventions and missing outcome data were considered to be at a low risk of bias.

**Figure 2:**
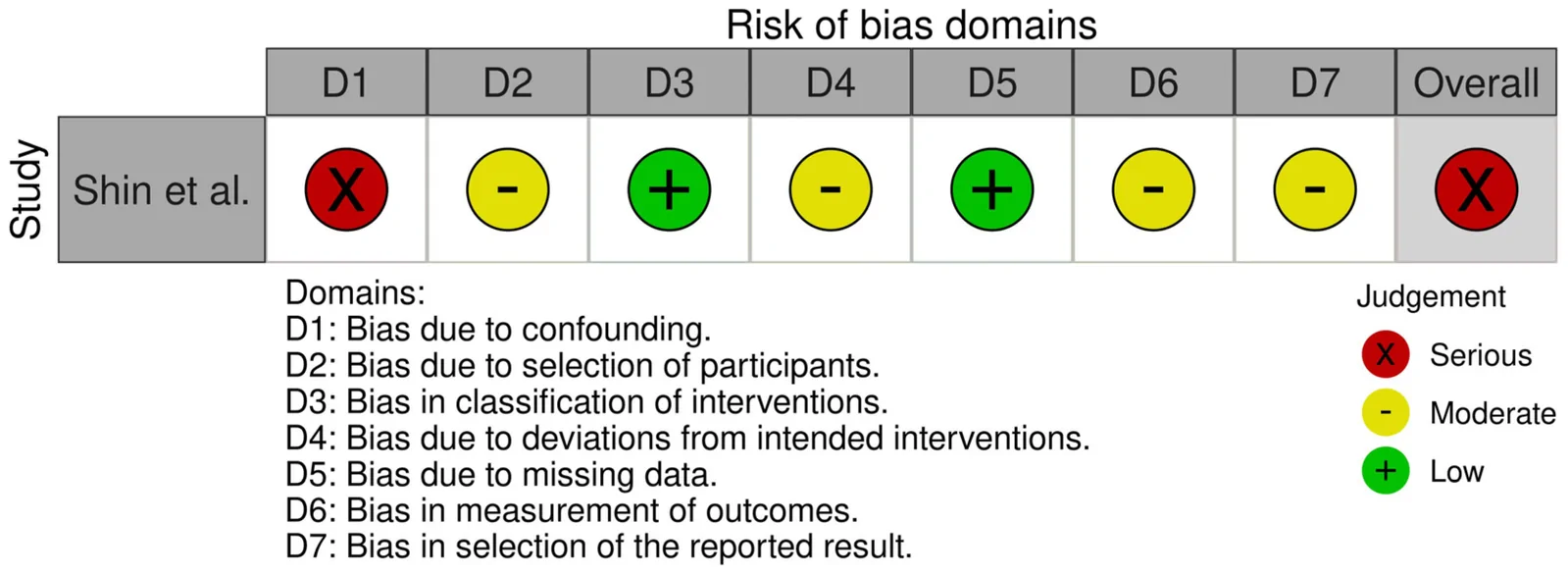
Risk-of-bias assessment of the non-randomized retrospective comparative study [24].

**Figure 3:**
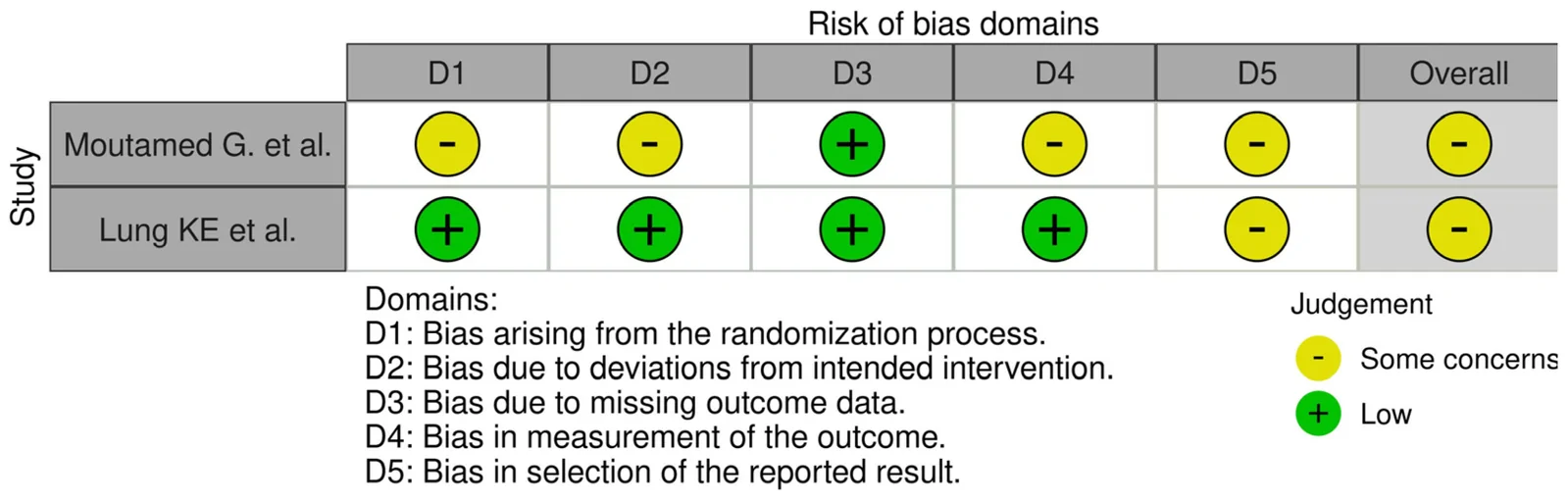
Risk-of-bias assessment of randomized controlled studies [22,23].

Visual summaries of the risk-of-bias assessments were generated using the robvis visualization tool and are presented in Figure 2 and Figure 3.

### 2.3. Characteristics of Included Studies

Three studies [22,23,24] were included in this systematic review. The included studies comprised randomized controlled trials and a retrospective comparative study evaluating the adjunctive use of botulinum toxin type A (BoNT-A) in patients undergoing orthognathic surgery. Although all studies investigated the perioperative application of BoNT-A, considerable heterogeneity was observed in the surgical procedures, injection sites, timing of administration, and evaluated clinical outcomes.

Two studies primarily investigated the potential effect of BoNT-A on postoperative skeletal or mechanical stability [22,24]. Shin et al. [24] evaluated bilateral BoNT-A injections into the masseter muscles in patients undergoing bilateral sagittal split ramus osteotomy for mandibular setback, assessing postoperative plate fracture and skeletal relapse using cephalometric angular measurements. Moutamed et al. [22] investigated preoperative BoNT-A administration into the anterior belly of the digastric muscle in patients undergoing mandibular advancement as part of bimaxillary orthognathic surgery, with postoperative skeletal relapse assessed using linear and angular cephalometric measurements.

In contrast, Lung et al. [23] focused on postoperative recovery following bilateral sagittal split osteotomy, evaluating the effect of preoperative BoNT-A administration on postoperative pain and opioid consumption. Thus, rather than examining a single uniform outcome, the included evidence addressed different potential clinical effects of perioperative BoNT-A administration, encompassing mechanical fixation-related complications, postoperative skeletal stability, and postoperative pain management.

Overall, the included studies allowed evaluation of BoNT-A as an adjunct to orthognathic surgery across three clinically relevant domains: mechanical stability, skeletal stability and relapse, and postoperative recovery. Due to heterogeneity in treatment protocols and outcome measures, the findings were considered individually rather than quantitatively pooled. The main characteristics and extracted outcomes of the included studies are presented in Table 1.

### 2.4. Mechanical Stability and Fixation-Related Outcomes

Mechanical stability and fixation-related outcomes were evaluated by Shin et al. [24] in patients undergoing BSSRO. The study compared patients receiving postoperative BoNT-A injections into the bilateral masseter muscles with patients who received no BoNT-A treatment. Plate fracture was observed less frequently in the BoNT-A group, with 2 of 16 fixation plates affected compared with 8 of 16 plates in the control group (*p* = 0.031). Postoperative skeletal changes were additionally assessed using the sella–nasion–B point (SNB) angle, mandibular plane angle, and gonial angle. No statistically significant between-group differences were identified in changes in these cephalometric parameters during follow-up (*p* > 0.05).

### 2.5. Skeletal Stability and Postoperative Relapse

The effect of BoNT-A on postoperative skeletal relapse following mandibular advancement was investigated by Moutamed et al. [22]. Patients received preoperative BoNT-A injections into the anterior belly of the digastric muscle or underwent the same orthognathic surgical treatment without BoNT-A administration. Skeletal stability was evaluated using postoperative cone-beam computed tomography and included linear measurements of the B-point and Pogonion in relation to reference planes, together with angular measurements including SNB, ANB, and Ar-Go/SN. A statistically significant reduction in anteroposterior relapse at the Pogonion relative to the coronal reference plane was reported in the BoNT-A group. For the remaining linear and angular measurements, no statistically significant differences between the groups were observed. Thus, the reported effect on postoperative relapse was evident for a specific linear skeletal parameter rather than consistently across all evaluated measurements.

### 2.6. Postoperative Pain, Analgesic Consumption, and Recovery

Postoperative pain and analgesic requirements were evaluated by Lung et al. [23] following mandibular advancement by BSSO. Patients receiving preoperative BoNT-A suggested lower mean preanalgesic pain scores compared with the placebo group (1.72 vs. 2.81; *p* < 0.05). Total opioid consumption during the 14-day postoperative assessment period was also lower in the BoNT-A group, with a mean of 4.9 opioid doses compared with 8.9 doses in the placebo group (*p* = 0.046). No statistically significant between-group differences were identified for postanalgesic pain, nonopioid analgesic consumption, or the frequency of postoperative muscle spasms. The study therefore identified differences in preanalgesic pain intensity and cumulative opioid use, whereas the remaining assessed recovery-related outcomes were comparable between groups.

## 3. Discussion

The present systematic review evaluated the available clinical evidence regarding the adjunctive use of botulinum toxin type A (BoNT-A) in orthognathic surgery. Although only three studies met the eligibility criteria, the available findings suggest that temporary modulation of perioperative muscle activity may influence mechanical stability, skeletal relapse, and postoperative recovery. However, substantial heterogeneity in surgical procedures, targeted muscles, injection timing and dosage, and evaluated outcomes limits direct comparison between studies and prevents definitive conclusions regarding the clinical effectiveness of BoNT-A.

Shin et al. [24] reported fewer fixation plate fractures following postoperative BoNT-A injection into the masseter muscles, although this was not accompanied by improved cephalometric skeletal stability. This finding may be explained by the temporary reduction in masticatory muscle activity and bite force produced by BoNT-A [25]. Previous clinical evidence has demonstrated significant reductions in electromyographic activity and maximum bite force following injections into the masseter and temporalis muscles, with these effects persisting for several weeks. Reduced muscular loading during the early healing period may therefore decrease mechanical stress on fixation hardware [26]. Nevertheless, the retrospective design, small sample size, and serious risk of bias of Shin et al. [24] should be considered when interpreting this finding. Furthermore, plate fracture was analyzed at the level of individual fixation plates rather than patients; therefore, potential within-patient clustering of bilateral fixation plates should be considered when interpreting the reported statistical significance.

Muscular traction may also contribute to postoperative skeletal relapse, particularly following mandibular advancement. Relapse after orthognathic surgery is multifactorial and may be affected by the magnitude and direction of surgical movement, fixation, condylar positioning, soft-tissue adaptation, and muscular forces. A previous systematic review suggested that changes in perimandibular muscle morphology and function may contribute to relapse following advancement bilateral sagittal split osteotomy [27]. Previous clinical observations involving the same muscle have also reported postoperative mandibular stability following BoNT-A administration [28,29]. However, because significant differences were not observed across all skeletal measurements in the included study, the current evidence is insufficient to conclude that BoNT-A consistently prevents postoperative relapse.

The findings of Lung et al. [23] suggest another potential application of BoNT-A in orthognathic surgery. Preoperative injection into the masseter and temporalis muscles was associated with lower preanalgesic pain scores and reduced opioid consumption during the first 14 postoperative days. Postoperative pain following orthognathic surgery can result in considerable analgesic requirements, particularly after mandibular and bimaxillary procedures [30]. The mean opioid consumption was 4.9 doses in the BoNT-A group compared with 8.9 doses in the placebo group, corresponding to approximately four fewer opioid doses during the 14-day postoperative period. Although this difference was statistically significant, the original study did not establish a predefined threshold for a clinically meaningful reduction in opioid consumption; therefore, its clinical significance should be interpreted cautiously. Importantly, the lower pain scores were observed for preanalgesic pain, whereas no significant between-group difference was found in postanalgesic pain. BoNT-A also did not significantly affect nonopioid analgesic consumption or muscle spasm frequency.

An important finding of the present review was the considerable variation in BoNT-A administration protocols. The included studies targeted the masseter, temporalis, or anterior belly of the digastric muscle and differed in both injection timing and total dose. These differences may reflect distinct therapeutic objectives: a reduction in masseter and temporalis activity may primarily decrease masticatory loading and postoperative pain, whereas targeting the digastric muscle may reduce muscular traction following mandibular advancement. Timing may also influence treatment effects, as the neuromuscular action of BoNT-A develops progressively after injection. Consequently, future studies should aim to establish standardized protocols according to the intended clinical outcome rather than considering perioperative BoNT-A administration as a single uniform intervention.

From a clinical perspective, the current evidence does not support routine perioperative use of BoNT-A in orthognathic surgery. Nevertheless, selected applications may warrant further investigation, particularly in patients at increased risk of excessive muscular loading of fixation systems, skeletal relapse, or substantial postoperative analgesic requirements. Future randomized controlled trials should investigate these indications separately and use standardized target muscles, doses, injection timing, and placebo-controlled designs. Outcomes should include patient-level fixation complications, standardized three-dimensional assessment of skeletal relapse, clearly differentiated pre- and postanalgesic pain outcomes, standardized measures of opioid consumption, and clinically relevant patient-reported outcomes. Adequate sample sizes and longer follow-up will also be necessary to determine whether potential early benefits translate into clinically meaningful and sustained outcomes.

Potential adverse effects of muscular unloading should also be considered. Although temporary reduction in muscle-generated forces may be beneficial during early healing, prolonged reduction in masticatory function has been associated with changes in mandibular bone. A systematic review and meta-analysis reported a reduction in mandibular cortical thickness following BoNT-A administration in human studies, although consistent effects on overall bone volume or density were not demonstrated [31]. No evidence of impaired osteotomy healing was reported in the studies included in the present review; nevertheless, the potential effects of repeated or prolonged muscle paralysis should be considered in future clinical investigations.

Several limitations of this systematic review should be acknowledged. Most importantly, only three studies met the eligibility criteria, with relatively small sample sizes and considerable methodological and clinical heterogeneity. The included studies differed in study design, direction of mandibular movement, targeted muscles, BoNT-A dosage and timing of administration, follow-up periods, and evaluated outcomes, which precluded quantitative synthesis of the results. Furthermore, one included study had a serious overall risk of bias, while some methodological concerns remained in the randomized studies. Additionally, the review was not prospectively registered and no formal review protocol was prepared, which reduces methodological transparency and should be considered when interpreting the findings. Nevertheless, the research question, eligibility criteria, outcomes of interest, and analysis approach were defined before the literature search and study-selection process and were not modified based on the identified studies. Furthermore, adverse events specifically attributable to BoNT-A administration were not systematically reported across the included studies, limiting conclusions regarding the safety of its perioperative use in orthognathic surgery. These limitations reduce the generalizability of the findings and prevent firm conclusions regarding the effectiveness of perioperative BoNT-A in orthognathic surgery.

## 4. Conclusions

The available evidence suggests that botulinum toxin type A may represent a promising adjunct to orthognathic surgery by temporarily reducing muscle activity during the postoperative healing period. Potentially favorable effects were observed including reduced mechanical loading of fixation hardware, which was associated with approved stability of selected skeletal parameters following mandibular advancement, and decreased postoperative pain and opioid consumption. However, these effects were not consistently demonstrated across all evaluated outcomes, and considerable heterogeneity was observed in surgical procedures, targeted muscles, injection protocols, and outcome assessment. Given the limited number of available studies and their methodological limitations, the current evidence is insufficient to support the routine use of BoNT-A in orthognathic surgery. Further well-designed randomized controlled trials with standardized injection protocols, larger patient populations, and longer follow-up periods are required to determine its clinical effectiveness and establish appropriate indications.

## 5. Materials and Methods

This systematic review was conducted in accordance with the Preferred Reporting Items for Systematic Reviews and Meta-Analyses (PRISMA) guidelines (Supplementary Document S2) [32]. The review was not prospectively registered, and a separate formal review protocol was not prepared. However, prior to conducting the literature search, the review question, PICO framework, eligibility criteria, outcomes of interest, and search strategy were defined a priori by the authors. The decision not to register the review was related to the exploratory nature of the initial literature assessment and the limited clinical evidence available on the adjunctive use of BoNT-A in orthognathic surgery. Importantly, the eligibility criteria and outcomes were not modified based on the results identified during the literature search. The review was designed to systematically identify and evaluate the available clinical evidence regarding the adjunctive use of botulinum toxin type A (BoNT-A) in patients undergoing orthognathic surgery. The primary research question was formulated as follows: in patients undergoing orthognathic surgery, does the adjunctive administration of BoNT-A, compared with orthognathic surgery without BoNT-A administration, influence clinical outcomes related to mechanical stability, skeletal stability, and postoperative recovery? To structure the research question and define the eligibility criteria, the PICO framework was applied.
The population(P)Patients undergoing orthognathic surgical procedures for the correction of dentofacial deformities.The intervention(I)Preoperative or postoperative administration of botulinum toxin type A (BoNT-A) as an adjunct to orthognathic surgeryThe control(C)Conventional orthognathic surgical treatment without adjunctive administration of botulinum toxin type A (BoNT-A).The outcomes(O)Clinical outcomes related to mechanical stability, skeletal stability/relapse, and postoperative recovery. Mechanical stability was defined as fixation-related integrity or complications (e.g., plate fracture), whereas skeletal stability referred to maintenance of the surgically achieved skeletal position over time; skeletal relapse was defined as postoperative change toward the preoperative skeletal position.

### 5.1. Search Strategy

A systematic electronic literature search was conducted independently by two reviewers in PubMed, the Cochrane Library, and Google Scholar. The final search was performed on 5 July 2026. The search strategy combined terms related to botulinum toxin type A with terms related to orthognathic surgery and relevant mandibular osteotomy procedures. The search syntax was adapted to the structure and search functionality of each database. Complete database-specific search strategies, including the exact search strings, Boolean operators, field restrictions, filters, and dates of execution, are provided in Supplementary Document S1. Google Scholar was included as a supplementary search source to increase search sensitivity and to identify potentially relevant studies that might not have been retrieved through the bibliographic databases. Given the limited reproducibility of Google Scholar searches, it was used as a complementary rather than primary search source, with potentially relevant records assessed according to the same predefined eligibility criteria. A predefined Boolean search strategy was applied across all databases. Following the removal of duplicate records, titles and abstracts were independently screened by both reviewers according to the predefined eligibility criteria. The full texts of potentially relevant publications were subsequently assessed to determine their final eligibility for inclusion.

A predefined search strategy combining keywords and Boolean operators was used to identify relevant publications. The following search terms were applied: (“botulinum toxin” OR “botulinum toxin type A” OR “BoNT-A” OR “BTX-A” OR “Botox”) AND (“orthognathic surgery” OR “mandibular advancement” OR “mandibular setback” OR “bilateral sagittal split osteotomy” OR “BSSO” OR “sagittal split ramus osteotomy” OR “SSRO”).

The literature search was restricted to articles published in English within the past ten years. No restrictions regarding the country of publication were applied.

The study selection process was conducted in two consecutive stages. Following the removal of duplicate records, the titles and abstracts of all identified publications were independently screened according to the predefined eligibility criteria. Potentially relevant studies subsequently underwent full-text assessment, and final eligibility was determined based on the established inclusion and exclusion criteria. In addition, the reference lists of the included studies were manually screened to identify potentially relevant publications not retrieved through the electronic database search.

Data extraction was performed independently by two reviewers using a predefined data-extraction framework. The following variables were collected from each included study: first author, publication year, country, study design, sample size, surgical procedure, direction of mandibular movement, timing of BoNT-A administration, target muscle, BoNT-A dose, comparator, follow-up period, evaluated outcomes, and principal quantitative findings. Extracted data were organized in a standardized table and cross-checked between reviewers. Any discrepancies were resolved through discussion and consensus.

### 5.2. Selection Criteria

Eligible publications included randomized controlled trials, controlled clinical trials, and observational studies evaluating the adjunctive administration of BoNT-A in patients undergoing orthognathic surgery. Studies were considered eligible if they directly compared patients receiving BoNT-A with a control group undergoing orthognathic surgery without BoNT-A administration and reported clinical outcomes related to mechanical stability, skeletal stability, or postoperative recovery. Only full-text articles published in English within the predefined ten-year period were considered for inclusion.

### 5.3. Exclusion Criteria

- Animal or in vitro studies;
- Case reports, case series, narrative reviews, systematic reviews, meta-analyses, conference abstracts, editorials, letters, or study protocols;
- Studies evaluating BoNT-A administration without an orthognathic surgical procedure;
- Studies investigating BoNT-A for conditions unrelated to the perioperative management of orthognathic surgery;
- Studies without a control group receiving orthognathic surgical treatment without BoNT-A administration;
- Studies that did not report outcomes related to mechanical stability, skeletal stability, or postoperative recovery;
- Non-English publications or studies for which the full text was unavailable.

### 5.4. Risk-of-Bias Assessment

The risk of bias was independently assessed by two reviewers using tools appropriate to the design of the included studies, with any disagreements resolved through discussion and consensus. Randomized controlled trials were evaluated using the Cochrane Risk of Bias 2 (RoB 2) tool version 22 August 2019 [33], which assesses five domains: bias arising from the randomization process, deviations from intended interventions, missing outcome data, measurement of the outcome, and selection of the reported result. Each domain was classified as a low risk of bias, some concerns, or a high risk of bias according to the RoB 2 assessment algorithm. The non-randomized retrospective comparative study was assessed using the Risk Of Bias In Non-randomized Studies of Interventions (ROBINS-I) tool [34]. ROBINS-I evaluates seven domains: bias due to confounding, selection of participants, classification of interventions, deviations from intended interventions, missing data, measurement of outcomes, and selection of the reported result. Domain-level and overall judgements were classified as a low, moderate, serious, or critical risk of bias or no information.

## Figures and Tables

**Table 1:** Characteristics and main outcomes of the studies included in the systematic review.

Study; Design	Country	Sample Size	Surgical Procedure	BoNT-A Timing, Site, and Dose	Comparator	Follow-Up/ Assessment Period	Principal Quantitative Findings	Outcomes Evaluated
Moutamed et al. [22], 2025 Randomized controlled trial	Egypt	*n* = 24 BoNT-A: 12; control: 12	Bimaxillary orthognathic surgery with BSSO for mandibular advancement in skeletal Class II patients	Preoperative; anterior belly of the digastric muscle; 50 U	Same surgical treatment without BoNT-A injection	1 week and 6 months postoperatively	Significantly lower anteroposterior relapse at the Pogonion relative to the coronal plane in the BoNT-A group; no significant differences in the other evaluated linear or angular skeletal measurements.	Skeletal relapse assessed by CBCT: linear B-point and Pogonion measurements relative to Frankfort horizontal and coronal planes; angular SNB, ANB, and Ar-Go/SN measurements
Lung et al. [23], 2026 Prospective randomized, double-blinded, placebo-controlled clinical trial	Canada	*n* = 40	BSSO for mandibular advancement	2 weeks preoperatively; bilateral temporalis and masseter muscles; 10 U per temporalis and 40 U per masseter per side (100 U total)	0.9% saline placebo injections	Daily postoperative assessment over 14 days for primary published outcomes	Preanalgesic pain: 1.72 vs. 2.81, *p* < 0.05; opioid consumption: 4.9 vs. 8.9 doses, *p* = 0.046 (BoNT-A vs. placebo); no significant differences in postanalgesic pain, nonopioid analgesic use, or muscle spasm frequency.	Pre- and postanalgesic pain intensity; opioid and nonopioid analgesic consumption; muscle spasm frequency; patient satisfaction
Shin et al. [24], 2018 Retrospective comparative study	Republic of Korea	*n* = 16 BoNT-A: 8; control: 8	BSSRO for mandibular setback; some patients also underwent Le Fort I osteotomy and/or genioplasty	Immediately postoperative; bilateral masseter muscles; 25 U per masseter (5 U at each of 5 sites; 50 U total)	Orthognathic surgery without BoNT-A injection	2 and 6 months; skeletal change assessed from immediate postoperative to 6 months	Plate fracture: 2/16 plates (BoNT-A) vs. 8/16 plates (control), *p* = 0.031; no significant between-group differences in skeletal relapse parameters.	Plate fracture; postoperative skeletal change using SNB angle, mandibular plane angle, and gonial angle

Abbreviations: ANB, A-point–Nasion–B-point angle; Ar-Go/SN, Articulare–Gonion/Sella–Nasion angle; BoNT-A, botulinum toxin type A; BSSO/BSSRO, bilateral sagittal split osteotomy/ramus osteotomy; CBCT, cone-beam computed tomography; SNB, Sella–Nasion–B-point angle.

## Data Availability

The original contributions presented in this study are included in the article/Supplementary Materials. Further inquiries can be directed to the corresponding author.

## Supplementary Materials

The following supporting information can be downloaded at https://www.mdpi.com/article/10.3390/toxins18090399/s1. Supplementary Document S1: Complete Database-Specific Search Strategies; Supplementary Document S2: PRISMA 2020 Checklist.

## Abbreviations

The following abbreviations are used in this manuscript:

ANB	A point–Nasion–B point angle
Ar-Go/SN	Articulare–Gonion/Sella–Nasion angle
BoNT-A	Botulinum toxin type A
BSSO	Bilateral sagittal split osteotomy
BSSRO	Bilateral sagittal split ramus osteotomy
BTX-A	Botulinum toxin type A
CBCT	Cone-beam computed tomography
EMG	Electromyography
PICO	Population, Intervention, Comparison, Outcome
PRISMA	Preferred Reporting Items for Systematic Reviews and Meta-Analyses
RCT	Randomized controlled trial
Rob2	Cochrane Risk of Bias 2 tool
ROBINIS-I	Risk Of Bias In Non-randomized Studies of Interventions
SNB	Sella–Nasion–B point angle
SSRO	Sagittal split ramus osteotomy
TMD	Temporomandibular disorder
U	Units
