# Supplementary material for: Adjunctive Use of Botulinum Toxin in Orthognathic Surgery: A Systematic Review of Current Evidence and Clinical Perspectives
Source: Toxins (Basel). 2026 Sep 18;18(9):399. doi: 10.3390/toxins18090399 (PMC13611581; doi:10.3390/toxins18090399)
Supplement: Supplementary file 1 [file toxins-18-00399-s001.zip › toxins-4533109-Supplementary document S1 Complete Database-Specific Search Strategie.pdf]

Supplementary Materials: Adjunctive Use of Botulinum Toxin in Orthognathic Surgery: A Systematic Review of Current Evidence and Clinical Perspectives

Audra Janovskienė, Justina Stučinskaitė-Maračinskienė, Žygimantas Petronis, Jan Pavel Rokicki, Dainius Razukevičius, Loreta Pilipaitytė

Supplementary Material. Complete Database-Specific Search Strategies

The literature search was conducted in PubMed, the Cochrane Library, and Google Scholar. The search combined terms related to botulinum toxin type A with terms related to orthognathic surgery. The exact search information is provided below.

1. PubMed

Search string:

> (“botulinum toxin” OR “botulinum toxin type A” OR “BoNT-A” OR “BTX-A” OR “Botox”) AND (“orthognathic surgery” OR “mandibular advancement” OR “mandibular setback” OR “bilateral sagittal split osteotomy” OR “BSSO” OR “sagittal split ramus osteotomy” OR “SSRO”)

Boolean operators: OR was used to combine synonymous terms within each concept; AND was used to combine the botulinum toxin and orthognathic surgery concepts.

Field restrictions: None.

Filters/restrictions: English-language publications; predefined ten-year publication period.

Date of execution: July 5, 2026.

2. Cochrane Library

Search string:

> (“botulinum toxin” OR “botulinum toxin type A” OR “BoNT-A” OR “BTX-A” OR “Botox”) AND (“orthognathic surgery” OR “mandibular advancement” OR “mandibular setback” OR “bilateral sagittal split osteotomy” OR “BSSO” OR “sagittal split ramus osteotomy” OR “SSRO”)

Boolean operators: OR was used to combine synonymous terms within each concept; AND was used to combine the botulinum toxin and orthognathic surgery concepts.

Field restrictions: None.

Filters/restrictions: English-language publications; predefined ten-year publication period.

Date of execution: July 5, 2026.

3. Google Scholar

Search string:

> (“botulinum toxin” OR “botulinum toxin type A” OR “BoNT-A” OR “BTX-A” OR “Botox”) AND (“orthognathic surgery” OR “mandibular advancement” OR “mandibular setback” OR “bilateral sagittal split osteotomy” OR “BSSO” OR “sagittal split ramus osteotomy” OR “SSRO”)

Boolean operators: OR was used to combine synonymous terms within each concept; AND was used to combine the botulinum toxin and orthognathic surgery concepts.

Field restrictions: None.

Filters/restrictions: English-language publications; predefined ten-year publication period.

Date of execution: July 5, 2026.

Additional search procedure

Following database searching and removal of duplicate records, titles and abstracts were screened according to the predefined eligibility criteria. The reference lists of included studies were also manually screened to identify potentially relevant publications not retrieved through the electronic database search.

Supplementary Material

Study	Reason for exclusion
Kang et al., 2019. *Botulinum toxin-A injection into the anterior belly of the digastric muscle for the prevention of post-operative open bite in class II malocclusions: a case report and literature review.[17].*	Case report; no eligible comparator group.
Coclici et al., 2021. *Ultrasound dimensional changes of the anterior belly of the digastric muscle induced by orthognathic surgery and botulinum toxin A injection in Class II malocclusion.[18].*	Case series; no eligible comparator group and outcomes not aligned with the predefined clinical outcome domains.
Ban et al., 2023. *Botulinum Toxin Injection into the Digastric Muscle: Current Clinical Use and a Report of Five Cases.[28].*	Case series/review; no eligible comparator group.
Guignardat et al., 2024. *Systematic review of the histological and functional effects of botulinum toxin A on masticatory muscles: Consideration in dentofacial orthopedics and orthognathic surgery.[20].*	Systematic review; no eligible direct comparative clinical study of perioperative BoNT-A in orthognathic surgery.
Val et al., 2026. *Botulinum toxin injections into masticatory muscles: A brief review on applications in dental occlusion management for orthognathic surgery and facial fractures.[21].*	Narrative review; not an eligible primary comparative clinical study.
